# Supplementary material for: SARS-CoV-2 infection is associated with intestinal permeability, systemic inflammation, and microbial dysbiosis in hospitalized patients
Source: Microbiol Spectr. 2024 Sep 30;12(11):e00680-24. doi: 10.1128/spectrum.00680-24 (PMC11537016; doi:10.1128/spectrum.00680-24)
Supplement: Figure S1 and Table S1 — Fig. S1: Hospitalization of COVID-19 patients and disease outcome. Table S1: Samples available for each group. [file spectrum.00680-24-s0001.pdf]

Supplementary Materials:

A

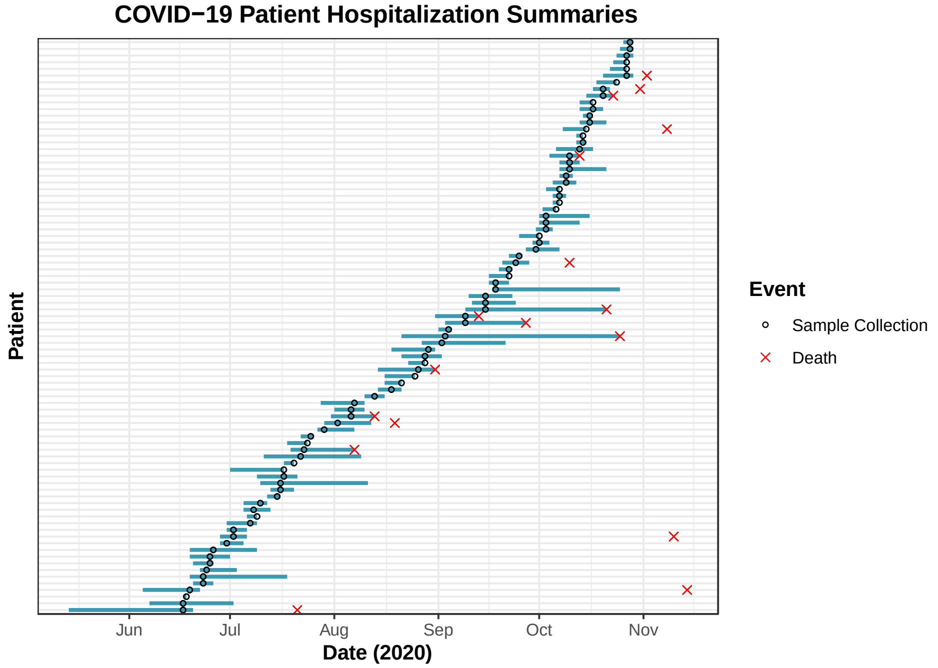

B

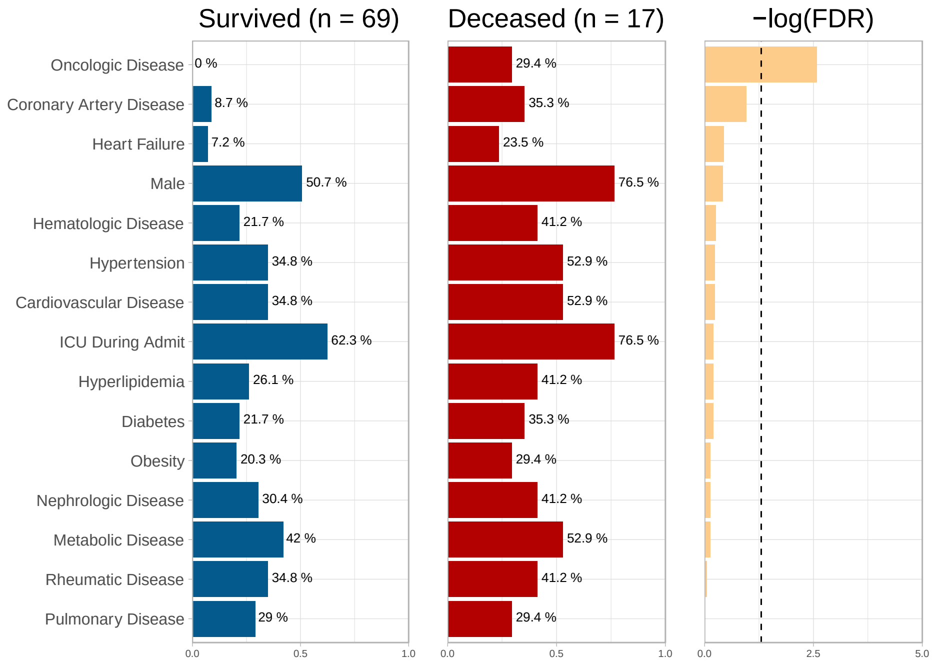

Supplemental Figure 1. Hospitalization of COVID-19 patients and disease outcome. (A) Summary of the COVID-19 patient hospitalizations. Blue bars represent the length of time each patient spent at the M Health Fairview Bethesda Hospital, with annotations for when samples were collected and when the patient died. **(B)** Prior history of comorbidities in COVID-19 patients who survived or died. Bars represent the percentage of patients in each group with a prior history of the comorbidity as well as the $-\log_{10}$(FDR adjusted p value) for the Chi-Squared or Fisher's exact test.

	Healthy	Survived	Deceased
Total patients enrolled (n)	12	69	17
Rectal swabs (n)	9	18	2
Oropharyngeal swabs (n)	10	50	14
Nasal swabs (n)	10	45	13
Plasma samples (n)	5	38	10

Supplemental Table 1. Samples available for each group.
